# New School Meal Regulations and Consumption of Flavored Milk in Ten US Elementary Schools, 2010 and 2013

**DOI:** 10.5888/pcd12.150163

**Published:** 2015-10-01

**Authors:** Bethany A. Yon, Rachel K. Johnson

**Affiliations:** Author Affiliation: Rachel K. Johnson, Department of Pediatrics, College of Medicine, and Nutrition and Food Sciences Department, University of Vermont, Burlington, Vermont.

## Abstract

Milk is a source of shortfall nutrients in children’s diets, but most children do not consume recommended amounts. We measured consumption of milk by elementary-schoolchildren (grades 3–5) in a diverse sample of schools before and after implementation of the US Department of Agriculture’s updated meal regulations requiring flavored milk to be fat-free. Flavored milk consumption did not change from 2010 to 2013; 52.2% of students in 2010 and 49.7% in 2013 consumed 7 ounces or more of an 8-ounce container. Updated regulations succeeded in lowering the amount of fat, added sugars, and calories in school milk but did not change overall milk consumption, thus improving children’s diet quality.

## Objective

Milk is a source of shortfall nutrients (vitamin D, calcium, and potassium) in children’s diets, supporting bone mass accumulation (1). Eating patterns are established early; children consuming soft drinks as early as age 5 years drink less milk as teenagers and consume more added sugars (2), which is associated with higher risk of obesity, diabetes (3), and coronary heart disease (4). Consuming milk, including flavored milk, is positively correlated with diet quality (5) and is not associated with excess weight gain (6). However, nearly 25% of children drink no milk on any given day (7). School meals are healthier than those sent from home (8); consuming milk with school lunch supports overall diet quality (5). Most (70%) elementary school students choose flavored milk over plain milk at school (9); children’s flavored milk consumption is not associated with higher intakes of added sugars (6). Banning flavored milk in school because of concerns about added sugars may lead to fewer children consuming milk (10). Children rejecting milk at school are unlikely to make up for the lost nutrients by consuming milk outside school (7). Updated US Department of Agriculture (USDA) regulations require all milk served in schools to be low-fat or fat-free (11). We assessed the consumption of flavored milk by schoolchildren after updated school meal regulations took effect.

## Methods

As part of a larger study evaluating the acceptance of lower-calorie (≤150 kcal per 8-oz serving) flavored milk, we asked 10 milk processors across the United States serving school districts in 23 states to identify public school districts using such flavored milk during the 2008–2009 or 2009–2010 school year. A purposive sample (n = 35) was selected for recruitment, and 22 school nutrition directors returned a signed informed consent form. We selected a convenience sample of 10 school districts in urban, suburban, or rural regions for a quasi-experimental plate-waste study (including 4 districts using standard flavored milk [>150 kcal per 8 oz]). Seven of the 10 districts are in the northeast (Massachusetts, New York, Rhode Island, and Vermont), and 3 are in the southern United States (Georgia and Texas). One elementary school serving grades 3 through 5 was randomly selected from each district for data collection in spring 2010 (baseline) and again in 2013 (follow-up) after flavored milk formulations changed to comply with updated USDA regulations (11).

At baseline and follow-up, milk containers were collected from students at the end of lunch and weighed to measure consumption. The grade, sex (identified by teachers), and container weights for each student were recorded. Descriptive analyses were used to describe school and student characteristics; we used χ^2^ analyses to examine proportional differences. Data on milk consumption were positively skewed, unresponsive to transformation, and thus reclassified into a binary variable (0–7 oz or >7 oz). Generalized linear mixed models (PROC GLIMMIX) were used to test differences in milk consumption while accounting for students nested within schools. A categorical variable for region was entered into the model as a proxy for differences between northeastern and southern schools in race/ethnicity and percentage of students eligible for free or reduced-price meals. Data were calculated as model-based least square means adjusted for region, grade, and sex. All analyses were 2-tailed tests performed using SPSS (version 21.0, IBM Corp) and SAS (version 9.4, SAS Institute, Inc). The University of Vermont Institutional Review Board approved this research as exempt.

## Results

The sample of schools was diverse in location, size, and racial/ethnic enrollment (Table 1). Flavored milks in all schools were fat-free during the 2012–2013 school year. Approximately 1.25 teaspoons of sugar per serving were removed from flavored milk in these schools; calories were reduced by up to 40 calories per 8-oz container. A total of 1,718 containers (n = 885 in 2010; n = 833 in 2013) of flavored milk (96% chocolate) were collected from students (52.5% boys) in 10 schools. Of these containers, 8.1% in 2010 and 10.8% in 2013 were unopened (χ^2^ = 3.6, *P* = .06). Compared with northeastern schools, a larger proportion of containers were unopened in southern schools (14.3% unopened in 2010, χ^2^ = 25.6, *P* < .001; 23.1% unopened in 2013, χ^2^ = 72.9, *P* < .001), where a higher percentage of students were eligible for free or reduced-priced meals.

**Table 1:** Demographic Characteristics of 10 Elementary Schools That Participated in Study of Flavored Milk Consumption and Flavored Milk Nutrition Profile, Before (Spring 2010) and After (Spring 2013) Updated USDA School Meal Regulations^a^^,^ b

Characteristic	Spring 2010	Spring 2013
**Elementary schools**		
School enrollment, mean (range), n	524 (184–1,009)	519 (222–972)
Student eligibility for free or reduced-priced school meals, mean (range), %	52.9 (3.6–88.9)	53.9 (5.6–87.7)
Student race/ethnicity, % (SD)		
White non-Hispanic	80.6 (22.6)	77.6 (21.6)
Black non-Hispanic	9.2 (15.3)	9.2 (15.3)
Hispanic	13.0 (36.3)	14.4 (35.4)
Asian	2.7 (2.7)	3.3 (3.2)
**Flavored milk nutrition profile per 8-oz container**		
kcal, range, n	150–170	110–130
Fat, %	0–1	0
Total sugars, range, g	22–27	18–22
Added sugars, range, tsp	2.5–3.75	1.5–2.5

Abbreviations: USDA, US Department of Agriculture; SD, standard deviation.

a USDA school regulations required flavored milk to be fat-free as of 2012–2013 school year.

b Study was conducted in 7 schools in Northeast (Massachusetts, New York, Rhode Island, Vermont) and 3 schools in the South (Georgia, Texas).

Milk consumption patterns differed by sex, grade, and region (Table 2). Overall, boys were more likely than girls to consume most (>7 oz) of their milk (odds ratio [OR], 1.53; 95% confidence interval [CI], 1.29–1.81; *P* < .001), this pattern was most evident in 2010 when 63.5% of boys and 53.6% of girls drank most of their milk (χ^2^ = 9.0, *P* = .003). Overall, students in grade 3 or 4 were more likely than students in grade 5 to consume most of their milk (OR, 1.45; 95% CI, 1.18–1.77; *P* = .001). Children in northern schools were slightly, but not significantly, more likely to consume most of their milk compared with children in southern schools over time (*P* = .12). After accounting for differences in region, sex, and grade, the likelihood that children would consume most of their milk did not change from 2010 to 2013 (OR, 0.90; 95% CI, 0.72–1.12; *P* = .35) (Table 2).

**Table 2:** Consumption of Flavored Milk by Students in Grades 3–5 With Lunch Before (Spring 2010)^a^ and After (Spring 2013)^b^ Updated USDA School Meal Regulations^c^^,^ d

Measure	Spring 2010 (n = 885 Milk Containers)	Spring 2013 (n = 833 Milk Containers)	*P* Value
**Flavored milk consumption, overall, unadjusted mean (SD), oz**	5.6 (2.8)	5.4 (2.9)	—e
**Children consuming >7 oz of 8-oz container of flavored milk, overall, mean^f^, %**	52.2	49.7	.35
**Children consuming >7 oz of 8-oz container of flavored milk, by region, unadjusted mean, %**			
Northeastern schools	67.0	67.0	.12
Southern schools	44.4	37.8	
**Children consuming >7 oz flavored milk, by sex, unadjusted mean, %**			
Boys	63.5	58.1	.003
Girls	53.6	54.8	

Abbreviation: USDA, US Department of Agriculture; SD, standard deviation.

a Milk profile in 2010: 150–170 kcal per 8-oz container, 0%–1% fat, 22–27 g total sugars, 2.5–3.75 tsp added sugars.

b Milk profile in 2013: 110–130 kcal per 8-oz container, fat-free, 18–22 g total sugars, 1.5–2.5 tsp added sugars.

c USDA school regulations required flavored milk to be fat-free as of 2012–2013 school year.

d Study was conducted in 7 schools in Northeast (Massachusetts, New York, Rhode Island, Vermont) and 3 schools in the South (Georgia, Texas).

e Could not be calculated because data were skewed.

f Means are model-based least squared means adjusted for region, grade, and sex.

## Discussion

This research showed that, among elementary schoolchildren selecting flavored milk with school lunch, consumption did not change from 2010 to 2013. Our study’s main strength is that we objectively measured milk consumption in a large, diverse sample of schools. The study had limitations. Although the flavored milks offered in schools participating in this study were reformulated, the nutrition profile of milk served varied among schools. Fewer children participated in school lunch programs under the new regulations; therefore our sample was smaller in 2013. Milk consumption may be influenced by other foods served in school. We collected data for only 1 day each year, and results may not be generalizable to schools in other parts of the country.

A recent American Academy of Pediatrics policy statement on school foods and beverages (12) supports the addition of small amounts of sugar to nutrient-dense foods to increase consumption by children. Updated USDA school meal standards have succeeded in removing fat and decreasing the amount of added sugars and calories in flavored milk served in schools without decreasing consumption. Public health and medical professionals can feel confident in encouraging children to drink milk, including flavored milk, with school meals because milk improves children’s nutrient intake and is low-fat (plain) or fat-free and limited in added sugars.
